# Self-reported non-adherence to P2Y12 inhibitors in patients undergoing percutaneous coronary intervention: Application of the medication non-adherence academic research consortium classification

**DOI:** 10.1371/journal.pone.0263180

**Published:** 2022-02-16

**Authors:** Yasushi Ueki, Thomas Zanchin, Sylvain Losdat, Alexios Karagiannis, Tatsuhiko Otsuka, George C. M. Siontis, Jonas Häner, Stefan Stortecky, Thomas Pilgrim, Marco Valgimigli, Stephan Windecker, Lorenz Räber

**Affiliations:** 1 Department of Cardiology, Bern University Hospital, Bern, Switzerland; 2 CTU Bern, University of Bern, Bern, Switzerland; 3 Cardiocentro, Lugano, Switzerland; University of Bologna, ITALY

## Abstract

**Aims:**

The Non-adherence Academic Research Consortium (NARC) has recently developed a consensus-based standardized classification for medication non-adherence in cardiovascular clinical trials. We aimed to assess the prevalence of NARC-defined self-reported non-adherence to P2Y12 inhibitors and its impact on clinical outcomes in patients undergoing percutaneous coronary intervention (PCI).

**Methods and results:**

Using a standardized questionnaire administered at 1 year after PCI, we assessed the 4 NARC-defined non-adherence levels including type, decision-maker, reasons, and timing within the Bern PCI registry. The primary endpoint was the patient-oriented composite endpoint (POCE) defined as a composite of death, myocardial infarction, stroke, and any revascularization at 1 year. The recommended P2Y12 inhibitor duration was 12 months. Among 3,896 patients, P2Y12 inhibitor non-adherence was observed in 647 (17%) patients. Discontinuation was permanent in the majority of patients (84%). The decision was mainly driven by a physician (94%), and rarely by patients (6%). The most frequent reason was risk profile change (43%), followed by unlisted reasons (25%), surgery (17%), and adverse events (14%). Non-adherence occurred early (<30 days) in 21%, late (30–180 days) in 45%, and very late (>180 days) in 33%. The majority of POCE events (n = 421/502, 84%) occurred during adherence to the prescribed P2Y12 inhibitor. Permanent discontinuation, doctor-driven non-adherence, and risk profile change emerged as independent predictors for POCE.

**Conclusions:**

In real-world PCI population treated with 1-year DAPT, non-adherence was observed in nearly one-fifth of patients. Non-adherence to P2Y12 inhibitors was associated with worse clinical outcomes, while the risk was related to underlying contexts.

**ClinicalTrials.gov identifier:**

NCT02241291.

## Introduction

Pharmacotherapy assumes a pivotal role to reduce morbidity and mortality from cardiovascular diseases. However, its benefits are attenuated by non-adherence to prescribed therapies [1–3]. While non-adherence provides important insights on drug tolerability, it is recognized as a common problem that significantly affects clinical outcomes and health care cost in daily clinical practice [4]. Medication adherence is not optimal even in well-controlled settings of clinical trials and approaches to collect and incorporate data on non-adherence have been heterogeneous across randomized clinical trials. Recently, the Non-adherence Academic Research Consortium (NARC) developed a consensus-based standardized classification and framework for medication non-adherence in cardiovascular clinical trials in 2018 [5]. In this consensus document, 4 level classifications of non-adherence have been proposed: the degree of over- or under-exposure to study medication; the decision process and circumstances; the clinical scenario; and timing relative to treatment initiationThe use of a P2Y12 inhibitor combined with aspirin (dual antiplatelet therapy [DAPT]) represents the standard of care for patients undergoing percutaneous coronary intervention (PCI), i.e. one of the most frequently performed invasive medical treatments worldwide [6]. Non-adherence to P2Y12 inhibitors may be associated with an increased risk of ischemic adverse events. The PARIS registry demonstrated that cardiac events depend on the reasons, timing and circumstances of unplanned DAPT cessation [7]. To date, the prevalence and potential clinical impact of non-adherence to P2Y12 inhibitors defined by the NARC classification have not been investigated. Therefore, we aimed to assess 1) the prevalence of NARC-defined non-adherence to P2Y12 inhibitors and 2) its impact on clinical outcomes using prospectively collected data from a large cohort of unselected, consecutive patients undergoing PCI.

## Method

### Study population and follow-up

The following patients were excluded from the Bern PCI registry (NCT02241291) for this analysis: treatment by balloon angioplasty alone, not discharged with a P2Y12 inhibitor or DAPT, oral anticoagulant at discharge, in-hospital death, and no information on P2Y12 inhibitor adherence.

Drug-adherence patterns were investigated as part of a pre-specified substudy of the prospective Bern PCI Registry conducted between 2011 and 2015. Patients were systematically and prospectively followed throughout 1 year to assess death, MI, stroke, revascularization, stent thrombosis (ST), bleeding complications, rehospitalization, and status of medical treatment. A questionnaire on the adherence to P2Y12 inhibitors was sent to patients at 1 year after index PCI (S1 File). These questionnaires were the basis to subsequently classify non-adherence events according to the NARC classification [5]. General practitioners, referring cardiologists, and patients were contacted as necessary for additional information on any non-adherence events. For patients with cardiovascular events or change in P2Y12 inhibitors, external medical records, discharge letters, and coronary angiography documentation were systematically collected and reviewed.

All patients provided written informed consent as part of a standard procedure. This prospective registry was approved by the institutional ethics committee (the Cantonal Ethics Committee [KEK] of the Canton of Bern), and all patients provided written informed consent.

### Procedures

PCI was performed in accordance with clinical practice guidelines [8]. DAPT consisting of aspirin and a P2Y12 inhibitor was initiated before, at the time, or immediately after the PCI procedure. Prasugrel was routinely used in patients presenting with ST-segment elevation myocardial infarction (MI) as of September 2009, and ticagrelor was routinely used in patients with Non-ST-segment elevation acute coronary syndrome as of November 2011. Aspirin at a dose of 100 mg once daily was continued indefinitely and the recommended duration of DAPT was 12 months [8]. The choice of a P2Y12 inhibitor was at the discretion of the treating physician.

### Clinical end points and definitions

#### Clinical end points

A clinical event committee consisting of 2 cardiologists (and a third referee in case of disagreement) adjudicated all events using original source documents. The primary endpoint of the study was the patient-oriented composite endpoint (POCE), defined as a composite of all-cause death, any stroke, any MI, or any revascularization at 12 months [9]. Secondary endpoints included major adverse cardiovascular endpoints (MACE), defined as a composite of cardiac death, MI, and stroke, the device oriented composite endpoints (DOCE), defined as a composite of cardiac death, target-vessel (TV) MI, and target lesion revascularization (TLR); all-cause death; cardiac death; any MI; TV-MI; any repeat revascularization; TLR; definite stent thrombosis (ST); stroke; any bleeding; Bleeding Academic Research Consortium (BARC) 3 or 5 bleeding, and BARC 2, 3, or 5 bleeding. Cardiac death was defined as any death caused by an immediate cardiac cause, procedure-related mortality, and death of unknown cause. Myocardial infarction (MI) was defined according to the modified historical definition [10]. Stent thrombosis was classified according to the Academic Research Consortium criteria [11]. Stroke was defined as rapid development of clinical signs of focal or global disturbance of cerebral function lasting >24 hours with imaging evidence of acute, clinically relevant brain lesion. Bleeding events were categorized according to the BARC classification [12].

#### Non-adherence definitions according to NARC

The NARC classification assesses four levels of non-adherence [5]; **Level 1 (type of non-adherence):** Type 0 (adherence to prescribed regimen) was defined as adherence to a P2Y12 inhibitor at discharge for 1 year and not fulfilling type 2 and 3 definitions. Type 1 (deviation from prescribed regimen) was not obtained within this registry. Type 2 (temporary discontinuation) was defined as temporary discontinuation of the prescribed P2Y12 inhibitor whereby the pharmacological half-life was taken into account (clopidogrel: 5 days; prasugrel: 7 days; ticagrelor: 3 days). Type 3 (permanent discontinuation) was defined as a permanent discontinuation of the prescribed P2Y12 inhibitor. We further differentiated Type 3 according to the current antiplatelet guideline [6]: “*Switch*” was defined as any change between prasugrel and ticagrelor. “*De-escalation*” was defined as any change from prasugrel or ticagrelor to clopidogrel. “*Escalation*” was defined as any change from clopidogrel to either prasugrel or ticagrelor. “*Discontinuation*” was defined as any definite permanent discontinuation without P2Y12 inhibitor replacement. **Level 2 (decision-maker responsible for non-adherence):** Investigator driven was not applicable in this registry study. Medical doctor driven was defined as change following the initiative of any physician. Patient driven was defined as change following the initiative of the patient. **Level 3 (reasons underlying non-adherence):** Risk profile change was defined as newly diagnosed/recognized medical condition, newly introduced/withdrawn concomitant medication, new information related to the study drug, or perception that medication not needed. Event was defined as adverse events or side effects related to a P2Y12 inhibitor. Surgery was defined as cardiac or non-cardiac surgery, or invasive procedures such as percutaneous coronary intervention and endoscopy. Unlisted was defined as reason not captured by the other categories. Logistic was defined as issues related to prescription, or complexity of the pharmacotherapy. Trauma was defined as temporary or permanent discontinuation as a direct result of trauma. **Level 4 (timing of non-adherence):** The original NARC classification leaves the timing of non-adherence open and we applied the following definition; Early was defined as any discontinuation within the first 30 days. Late was defined as any discontinuation between days 31–180 days. Very late was defined as any discontinuation after 180 days since baseline until one year. In case of more than one non-adherence categories were fulfilled, a hierarchical approach was applied, i.e. the more serious non-adherence event (e.g. permanent had priority over temporary discontinuation) was considered representative in the individual patient. We further classified patients with non-adherence into 3 groups according to the PARIS registry classification: (1) patient- or event-driven, (2) surgery-driven not fulfilling (1), (3) doctor driven not fulfilling (1) and (2) [7].

### Statistical analysis

Variables were summarized as mean ± standard deviation (SD) or as counts (percentages), and were compared between groups using Student’s t tests, Chi-square tests or Fischer’s exact tests, as appropriate. Kaplan-Meier cumulative event curves were constructed for time-to-event variables and compared using the log-rank test. Cox regression analysis was performed to test the prognostic significance of non-adherence. Non-adherence to P2Y12 inhibitors was entered to the model as a time-updated variable in order to consider a time-dependent manner of non-adherence during follow-up period (e.g. permanent discontinuation at 1 month: adherence between 0–1 month and non-adherence between 1–12 months) and adjusted by clinically important variables reported by previous studies including age, female sex, diabetes mellitus, estimated glomerular filtration rate, peripheral artery disease, myocardial infarction at presentation, cardiogenic shock, chronic obstructive lung disease, and history of cancer, history of PCI, and use of new generation drug eluting stents (DES). We calculated the estimated percentage of all POCE attributed to non-adherence as follows: the expected number of POCE was the observed number divided by hazard ratio (HR) [13]. The absolute attributable risk was the observed minus expected number of POCE. The percentage attributable risk was calculated as the absolute attributable risk expressed as a percentage of all POCE. P-values were two-tailed and considered under statistically significant at 0.05 threshold in all analyses. Statistical analyses were performed in STATA 15.

## Results

### Patient population

**Fig 1:**
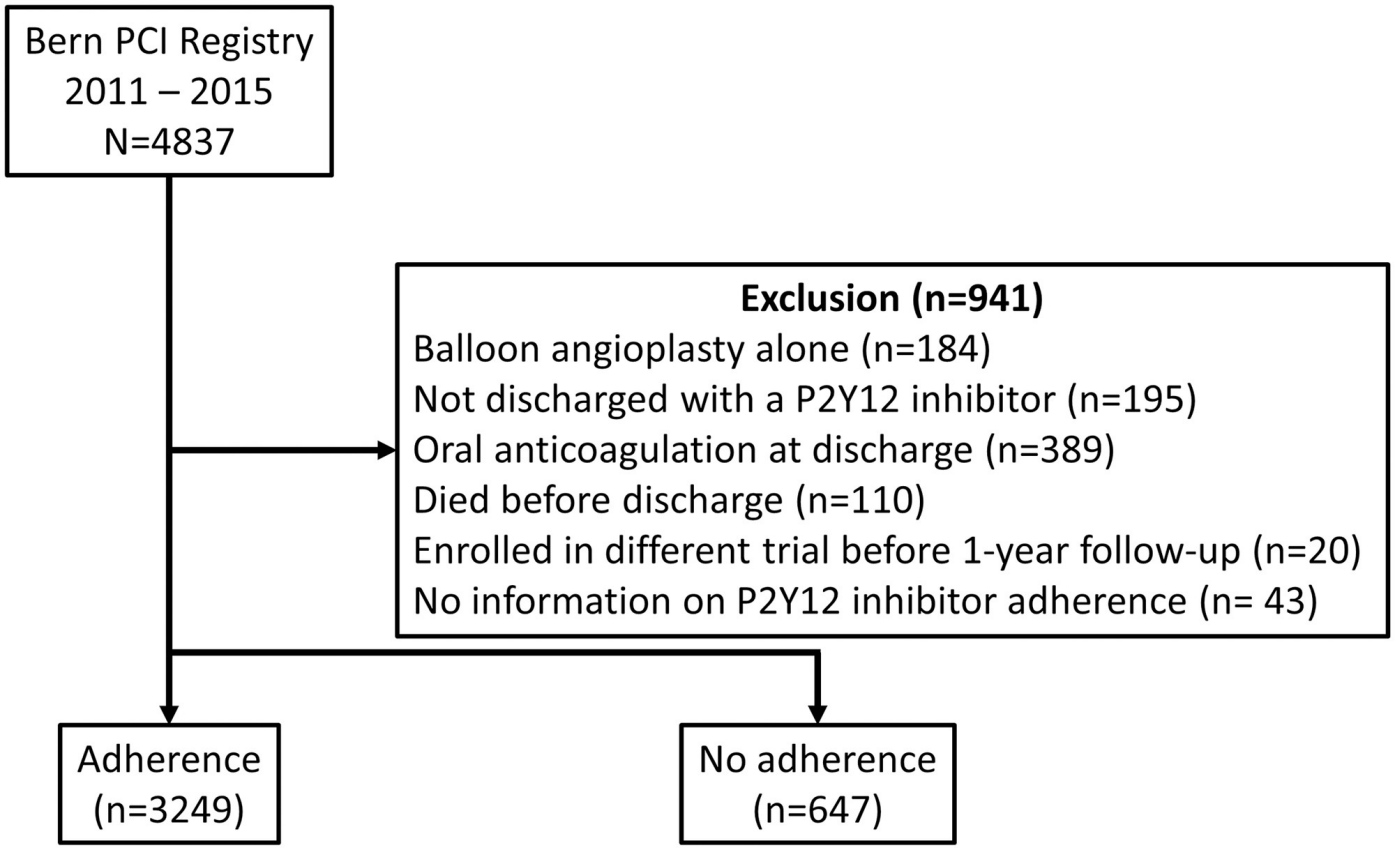
Patient flow. DAPT = dual antiplatelet therapy, PCI = percutaneous coronary intervention.

Of 4,837 consecutive patients prospectively enrolled into the Bern PCI Registry between 2011 and 2015, 3,896 patients were analyzed for the present study with complete follow-up available in 3667 (94.1%) patients at 1 year. Patients were excluded in case of balloon angioplasty without stent implantation (n = 184), absence of P2Y12 inhibitor at time of discharge (n = 195), any oral anticoagulant at time of discharge (n = 389), death before discharge (n = 110), enrolment in different trials before 1 year follow-up (n = 20), or no information on P2Y12 inhibitor adherence (n = 43) throughout one year (Fig 1).

### NARC-defined non-adherence

**Fig 2:**
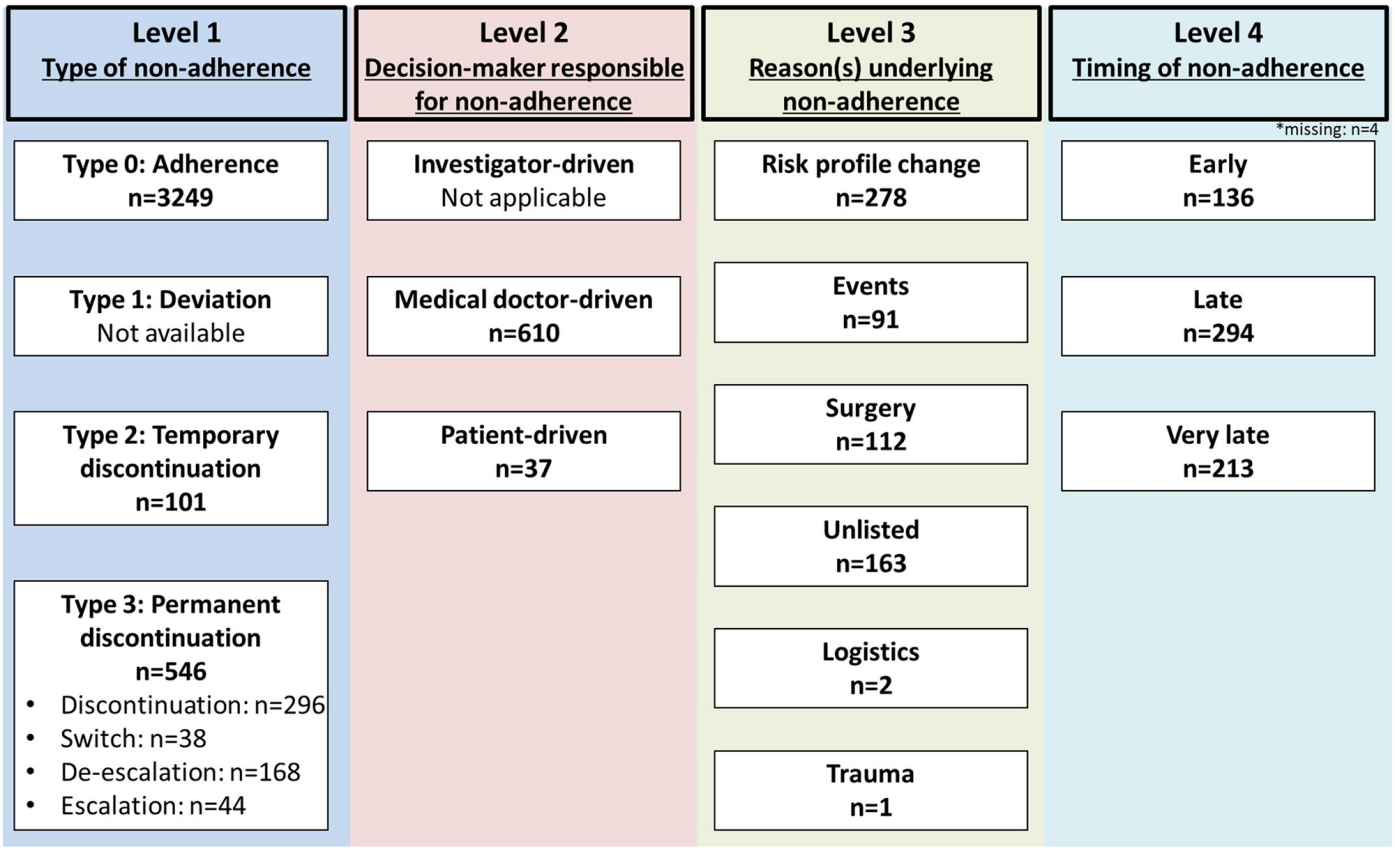
Overview of non-adherence according to NARC classification. NARC = non-adherence academic research consortium.

Fig 2 shows the prevalence of non-adherence to P2Y12 inhibitors according to the NARC classification (detailed breakdown in S1 Table). Level 1: A total of 647 (17%) patients fulfilled non-adherence criteria within 1-year of follow-up. Among those, the majority of patients (n = 546, 84%) were categorized as permanent discontinuation (discontinuation [n = 296, 54%], de-escalation [n = 168, 31%], escalation [n = 44, 8%] and switch [n = 38, 7%]), while temporary discontinuation occurred in the minority (n = 101, 16%). Level 2: Non-adherence was most frequently driven by the treating physician (n = 610, 94%), while only few were patient-driven (n = 37, 6%). Level 3: The most frequent reason for non-adherence was a change in risk profile (n = 278, 43%), followed by unlisted reasons (n = 163, 25%), surgical interventions (n = 112, 17%), adverse events (n = 91, 14%), logistic issues (n = 2, 0%), or trauma (n = 1, 0%). Level 4: non-adherence occurred early in 136 (21%), late in 294 (45%), and very late in 213 (33%). Median (25^th^ and 75^th^ percentile) days until P2Y12 non-adherence occurrence was 94 (30, 210) days (S1 Fig). Multiple non-adherence episodes occurred in 106 (16.3%) patients.

### Baseline characteristics

Clinical and procedural characteristics and medication status are summarized in Table 1. Patients with non-adherence were older, more likely to suffer from renal failure and anemia, and had a higher PRECISE-DAPT score compared to adherent patients. There were no significant differences related to procedural characteristics including stent length, stent diameter, and PCI complexity. In patients with non-adherence, prasugrel was less frequently and ticagrelor more frequently used.

**Table 1:** Baseline characteristics.

	Non-adherence	Adherence	P value
	(n = 647)	(n = 3249)	
Age (years)	68.7 ± 11.8	67.0 ± 12.2	0.002
Female	172 (26.6%)	849 (26.1%)	0.807
Current smoker	164 (25.8%)	935 (29.2%)	0.084
Hypertension	440 (68.3%)	2222 (68.8%)	0.852
Dyslipidemia	423 (65.5%)	2162 (66.9%)	0.493
Diabetes mellitus	145 (22.4%)	721 (22.2%)	0.918
Insulin use	61 (42.4%)	249 (34.9%)	0.106
Renal failure (eGFR<60ml/min/1.73m^2^)	177 (30.7%)	683 (23.1%)	<0.001
Anemia (men<13.0 g/dL, women<12.0 g/dL)	158 (29.4%)	627 (22.5%)	0.001
Chronic obstructive lung disease	38 (5.9%)	181 (5.6%)	0.779
History of malignancy	80 (12.4%)	333 (10.3%)	0.124
Peripheral arterial disease	65 (10.1%)	255 (7.9%)	0.071
History of Cerebrovascular Accident (Stroke/TIA)	59 (9.1%)	233 (7.2%)	0.102
Previous PCI	157 (24.3%)	688 (21.2%)	0.085
Left ventricular ejection fraction	52.8 ± 14.0	54.0 ± 13.4	0.041
PRECISE-DAPT score	20.4 ± 10.9	19.0 ± 11.2	0.028
Clinical indication for PCI			
Chronic coronary syndrome	253 (39.1%)	1350 (41.6%)	0.256
Unstable Angina	28 (4.3%)	192 (5.9%)	0.135
Non-ST elevation myocardial infarction	190 (29.4%)	829 (25.5%)	0.045
ST elevation myocardial infarction	176 (27.2%)	878 (27.0%)	0.923
Number of stents			
1	270 (41.7%)	1300 (40.0%)	0.430
2	181 (28.0%)	991 (30.5%)	0.205
≥3	196 (30.3%)	958 (29.5%)	0.706
Stent type			
New generation drug eluting stent	599 (92.6%)	3147 (96.9%)	<0.001
1st generation drug eluting stent	0 (0.00%)	8 (0.3%)	0.367
Bare metal stent	45 (7.0%)	75 (2.3%)	<0.001
Total device length (mm)	43.7 ± 29.1	42.6 ± 27.6	0.365
Mean stent diameter (mm)	3.0 ± 0.5	3.0 ± 0.5	0.833
Multivessel treatment	172 (26.6%)	856 (26.4%)	0.922
Bifurcation stenting	51 (7.9%)	216 (6.7%)	0.268
Chronic total occlusion	29 (4.5%)	139 (4.3%)	0.832
In-stent restenosis	34 (5.3%)	221 (6.8%)	0.164
Medication at discharge			
Clopidogrel	276 (42.7%)	1502 (46.2%)	0.101
Prasugrel	118 (18.2%)	778 (24.0%)	0.001
Ticagrelor	253 (40.5%)	969 (31.7%)	<0.001
Statin	584 (90.3%)	3028 (93.2%)	0.013

Values are n (%) or mean±SD.

eGFR = estimated glomerular filtration rate, PCI = percutaneous coronary intervention, TIA = transient ischemic attack.

### Clinical outcomes

**Fig 3:**
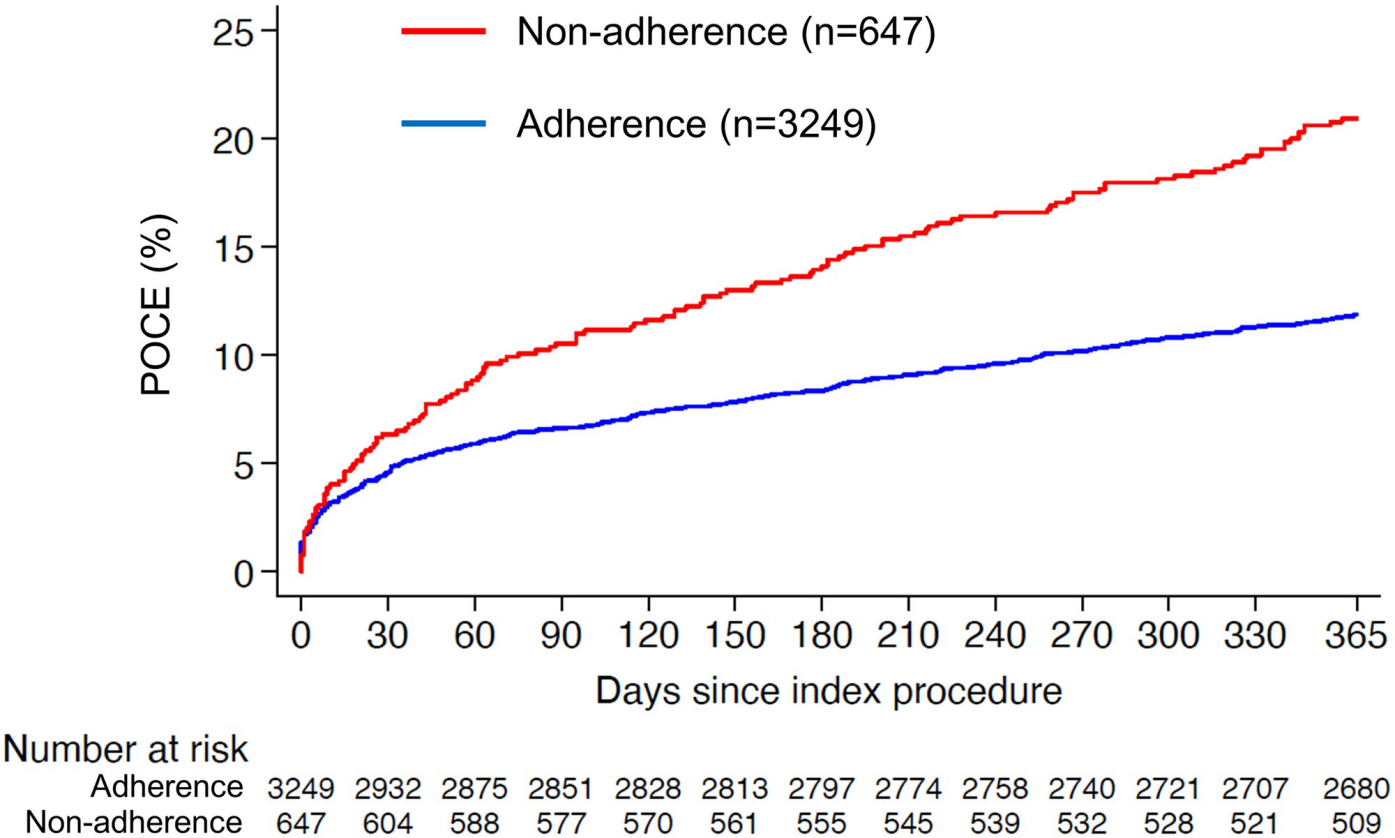
Kaplan-Meier cumulative event curve for POCE at 1 year. POCE = patient-oriented composite endpoints.

Clinical outcomes throughout 1-year are summarized in Table 2. Compared to patients with adherence, those with non-adherence had an increased risk of POCE (20.9% vs. 11.3%, P<0.001) (Fig 3), mainly driven by higher rates of MI (6.0% vs. 3.3%, P = 0.002), revascularization (12.5% vs. 6.2%, P<0.001), and stroke (3.1% vs. 0.7%, P<0.001). There was no significant difference in rates of definite stent thrombosis (1.2% vs. 0.8%, P = 0.356) between groups. Patients with non-adherence had a 4-fold higher risk of BARC 3 or 5 bleeding compared with those without (9.3% vs. 1.9%, P<0.001). Clinical outcomes occurred during adherence and non-adherence among patients with non-adherence (n = 647) are summarized in S2 Table. We confirmed that, among patients with non-adherence, adverse events indeed occurred more frequently during non-adherence.

**Table 2:** Event rate at 1 year.

	Non-adherence	Adherence	P value
	(n = 647)	(n = 3249)	
POCE (death, MI, revascularization, or stroke)	135 (20.9%)	367 (11.3%)	<0.001
MACE (cardiac death, MI, or stroke)	76 (11.7%)	204 (6.3%)	<0.001
DOCE (cardiac death, TV-MI, or TLR)	69 (10.7%)	223 (6.9%)	0.003
Death	36 (5.6%)	139 (4.3%)	0.275
Cardiac death	20 (3.1%)	88 (2.7%)	0.765
Myocardial infarction	39 (6.0%)	107 (3.3%)	0.002
Target vessel myocardial infarction	24 (3.7%)	85 (2.6%)	0.165
Periprocedural myocardial infarction	7 (1.1%)	47 (1.4%)	0.465
Spontaneous myocardial infarction	33 (5.1%)	61 (1.9%)	<0.001
Any Revascularization	81 (12.5%)	201 (6.2%)	<0.001
Target lesion revascularization	42 (6.5%)	102 (3.1%)	<0.001
Stroke	20 (3.1%)	23 (0.7%)	<0.001
Definite stent thrombosis	8 (1.2%)	27 (0.8%)	0.356
Any bleeding	93 (14.4%)	105 (3.2%)	<0.001
BARC (3, 5) bleeding	60 (9.3%)	61 (1.9%)	<0.001
BARC (2, 3, 5) bleeding	89 (13.8%)	102 (3.1%)	<0.001

Values are n (%).

BARC = bleeding academic research consortium, DOCE = device-oriented composite endpoints, MI = myocardial infarction, POCE = patient-oriented composite endpoints, TLR = target lesion revascularization, TV-MI = target-vessel myocardial infarction.

**Fig 4:**
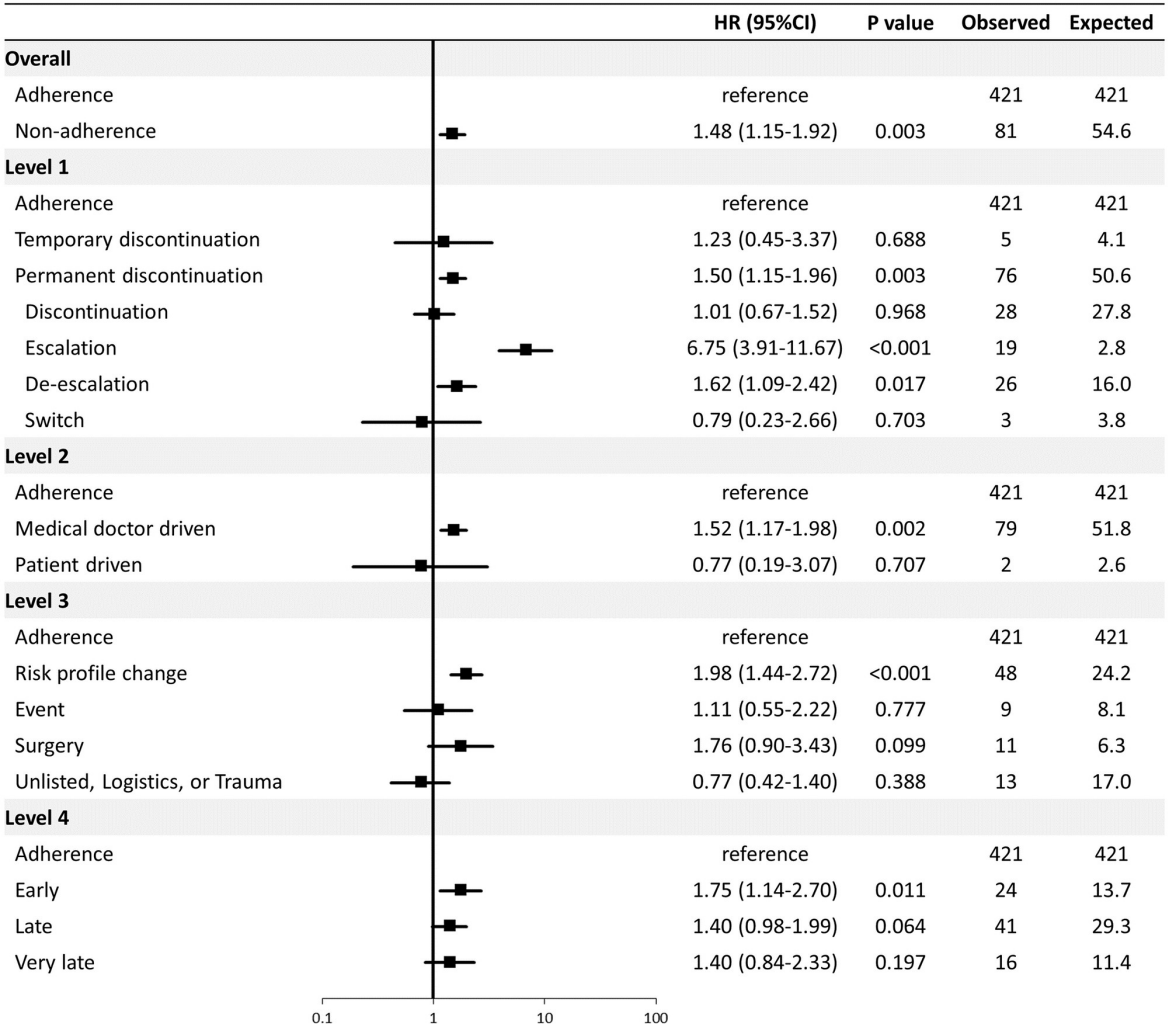
Multivariable Cox analysis for POCE at 1 year according to non-adherence levels. Of the study patients, 90.4% (3525 of 3896 patients) were included in the multivariable models. The following covariates were entered in the models: age, female sex, diabetes mellitus, eGFR, peripheral artery disease, myocardial infarction at presentation, cardiogenic shock, chronic obstructive lung disease, history of cancer, history of PCI, and use of new generation DES. CI = confidence interval, DAPT = dual antiplatelet therapy, DES = drug eluting stent, eGFR = estimated glomerular filtration rate, HR = hazard ratio, PCI = percutaneous coronary intervention.

The majority of POCE events (421; 84%) occurred during adherence to the prescribed P2Y12 inhibitor. During P2Y12 inhibitor non-adherence, 81 (16%) events were observed compared with an expected 54.6 events (81 events / HR 1.48) if a patient’s risk had been the same as adherence, which corresponds to an excess of 26.4 events (i.e. absolute attributable risk: the observed [81 events] minus expected number [54.6 events]). Among the overall incidence of POCE, 5.3% (26.4/502 events) can be statistically attributed to non-adherence to P2Y12 inhibitors. Fig 4 summarizes results of the multivariable Cox analysis for POCE according to the level of non-adherence and considering whether POCE occurred during non-adherence or not. Permanent discontinuation (HR 1.50, 95% confidence interval [CI] 1.15–1.96, P = 0.002) mainly driven by escalation (HR 6.75, 95% CI 3.91–11.67, P<0.01) and de-escalation (HR 1.62, 95% CI 1.09–2.42, P = 0.017) (Level 1), medical doctor-driven non-adherence (HR 1.52, 95% CI 1.17–1.98, P = 0.002) (Level 2), non-adherence due to risk profile change (HR 1.98, 95% CI 1.44–2.72, P<0.001) (Level 3), and non-adherence occurring during the early phase (HR 1.75, 95% CI 1.14–2.70, P = 0.011) (Level 4) emerged as independent predictors of POCE. Results remained consistent with use of a more restrictive endpoint (MACE: a composite of cardiac death, MI, and stroke) (S2 Fig). As an explanatory analysis, we further classified patients according to clinical indication for PCI (acute coronary syndrome [ACS] or chronic coronary syndrome [CCS]). Consistent results were observed in ACS patients, while non-adherence did not emerge as an independent predictor for POCE in patients with CCS (S3 and S4 Figs).

### PARIS category

Baseline characteristics and event rates according to PARIS category are summarized in S3–S5 Tables and S5 and S6 Figs. Multivariate Cox analysis according to PARIS category demonstrated that medical-doctor driven non-adherence was associated with an increased risk of POCE (HR 1.56, 95% CI 1.17–2.08, P = 0.003), while patient- or event-driven non-adherence (HR 1.01, 95% CI 0.52–1.94, P = 0.985) and surgery-driven non-adherence (HR 1.78, 95% CI 0.91–3.47, P = 0.090) were not (S7 Fig).

## Discussion

The present study is the first to apply the NARC classification for quantification of P2Y12 inhibitor non-adherence using a prospective questionnaire-based assessment of non-adherence patterns obtained from a large cohort of unselected, consecutive patients undergoing PCI treated with 1-year DAPT as standard of care. The main findings can be summarized as follows:

1. Non-adherence to P2Y12 inhibitors according to the NARC classification were observed among 17% of patients within the first year after PCI.
2. Non-adherence was most frequently permanent, driven by physicians in the majority of cases owing to changes in risk profile change of affected patients.
3. Most POCE events (84%) occurred while patients were adherent to the prescribed P2Y12 inhibitor.
4. Although the overall impact was modest, non-adherence was associated with impaired clinical outcomes and the risk varied according to type of non-adherence, decision makers, reasons, and timing.

There has been substantial variation in the definition of medication non-adherence. The NARC classification was established to categorize the complex nature of medication non-adherence in a standardized fashion. The current study demonstrated that this multilayered system to provide detailed information on non-adherence was applicable to P2Y12 inhibitors in the real-world PCI population with the exception of deviations from the prescribed regimen (Level 1, type 1), which would requires a highly reliable method to capture adherence pattern such as electronic medication monitors.

While a previous meta-analysis synthesizing data on 376,162 patients from 20 observational studies assessing adherence to cardiovascular drugs reported that a summary estimate of the prevalence of poor adherence on multiple cardiovascular drugs was 43% as assessed by pharmacy refill data [14], NARC-defined non-adherence to P2Y12 inhibitors occurred in 17% of patients undergoing PCI in the current study. This high adherence appears to be related to the proper implementation of recommended therapies by general practitioners, the precise understanding of the rationale for DAPT by patients, and the good tolerability of antiplatelet drugs. In the PARIS registry, any DAPT cessation was confirmed in 53% of patients undergoing PCI with stent implantation, although this was within a timeframe of 2-year follow-up [7].

In line with previous finding of the PARIS registry, most of POCE (84%) occurred while patients were adherent to the prescribed P2Y12 inhibitor, including the majority of stent thrombosis events (30 out of 35 events, 86%). Consequently, the risk attributable to POCE in relation to non-adherence was modest (estimated 5%), which may be at least partly due to improved efficacy and safety of new generation DES. In this regard, our data are fully in line with the safety of short-term (1–3 months) DAPT after PCI with new generation DES [15–19].

In the current study, escalation and de-escalation (Level 1), medical doctor-driven (Level 2), and risk-profile change (Level 3) emerged as an independent predictor for POCE. There were significant overlaps among these types of non-adherence (S1 Table). Almost all de-escalation/escalation were initiated by physicians (n = 210/212, 99%) and mainly due to a change in risk profile (n = 134/212, 63%). De-escalation/escalation and risk profile change accounted for 34% (n = 210/610) and 45% (n = 276/610) of medical doctor-driven non-adherence, respectively. Among non-adherence due to risk profile change, 48% (n = 134/278) were de-escalation/escalation and 99% (n = 276/278) were initiated by medical doctors. In routine clinical practice, it is common that physicians change the prescribed P2Y12 inhibitor (i.e. de-escalation/escalation) due to newly recognized medical conditions (e.g. cancer) or newly introduced medications (e.g. introduction of oral anticoagulation) during the course after index PCI. Although multivariable adjustment was performed using relevant clinical factors available at baseline, this cannot correct for confounding factors that newly arise after the index PCI and subsequently lead to non-adherence. This may be exemplified considering an increased hazard of escalation and de-escalation, which in itself may not impact the risk, but rather represents a surrogate marker of risk (e.g. recurrent ischemic event or bleeding). Collectively, our findings suggest that the clinical context underlying non-adherence represents the key driver of the risk hazards rather than doctor’s decision itself.

In variance to our study, the PARIS registry has reported that medical doctor-driven non-adherence was associated with a lower risk of 2-year MACE [7]. In the PARIS registry in which 35% of patients were treated with bare metal stent or 1st generation DES, the reference standard was adherence to DAPT for 2 years. Majority of physician-guided discontinuations occurred around 1 year (the mean duration of sustained DAPT in patients with physician-guided discontinuation: 382±169 days), which appears to be more appropriate management rather than 2-year DAPT [6], thereby accounting for lower adverse events after discontinuation. With regard to non-adherence driven by patient or events, no significant association with clinical outcomes was observed in the current study, while the PARIS registry showed that the disruption driven by patients or bleeding was associated with a substantially increased risk of MACE. This difference may be partly attributable to the limited number of patients in this category (patient-driven: n = 37, event-driven: n = 91) in the current study.

There was a clear signal that early non-adherence (<30 days) was associated with the highest risk of POCE with an attenuation over time, especially among patients with ACS. Similarly, previous studies have demonstrated that the risk of non-adherence to DAPT was highest within the first 6 months after stent implantation and was attenuated beyond this time point [7, 20]. In the current study, however, this trend was not observed in CCS patients. Although the results obtained from sub-group analysis need careful interpretation, clinical presentation (i.e. ACS vs. CCS) may be an important determinant when considering the time-related risk of non-adherence.

## Limitations

First, the single-center design of this study may limit the generalizability of our findings. Second, some of the NARC classification levels were not applicable or modified according to data availability in our database. Third, data on adherence to aspirin was not available in the current study. Fourth, the reference standard in the current study was adherence to the prescribed P2Y12 inhibitor for 12 months, which is different from the latest guideline recommendation (i.e. 6 months) [6]. Fifth, adherence pattern were collected based on questionnaires at 1 year after PCI, which may leads to underreporting by recall bias and particularly render information on patient-driven adherence less reliable [21]. Although we were able to assess and characterise in detail non-aherence events in 17% of patients, if non-adherence was captured with more sophisticated methods (e.g. electronic medication monitors), the prevalence and patterns may have been different from that in the current study. Further investigations are required in this regard.

## Conclusion

In real-world PCI population treated with 1-year DAPT as a standard care, non-adherence was observed in nearly one-fifth of patients and most of clinical events occurred during adherence to P2Y12 inhibitors. Non-adherence to P2Y12 inhibitors was associated with worse clinical outcomes in patients undergoing PCI, while the risk was related to underlying contexts and timing.

## Supporting information

S1 Fig Cumulative frequency curve for non-adherence. (DOCX)

S2 Fig Multivariate Cox analysis for a composite of cardiac death, MI, and stroke according to non-adherence level. (DOCX)

S3 Fig Multivariable Cox analysis for POCE at 1 year according to non-adherence levels among ACS patients. (DOCX)

S4 Fig Multivariable Cox analysis for POCE at 1 year according to non-adherence levels among CCS patients. (DOCX)

S5 Fig Cumulative frequency curve for P2Y12 non-adherence according to PARIS category. (DOCX)

S6 Fig Kaplan-Meier cumulative event curve for POCE according to PARIS category. (DOCX)

S7 Fig Multivariate Cox analysis according to PARIS category. (DOCX)

S1 Table Prevalence of non-adherence according to NARC classification. (DOCX)

S2 Table Clinical outcomes occurred during adherence and non-adherence among patients with non-adherence. (DOCX)

S3 Table Baseline characteristics according to PARIS category. (DOCX)

S4 Table Non-adherence according to PARIS category. (DOCX)

S5 Table Event rate at 1 year according to PARIS category. (DOCX)

S1 File Questionnaire on the adherence to P2Y12 inhibitors. (DOCX)

## Abbreviations

BARC: bleeding academic research consortium
CAD: coronary artery disease
CKD: chronic kidney disease
DAPT: dual antiplatelet therapy
DES: drug eluting stent
DOCE: device oriented composite endpoint
MACE: major adverse cardiovascular endpoint
MI: myocardial infarction
NARC: non-adherence academic research consortium
PCI: percutaneous coronary interventions
POCE: patient-oriented composite endpoint
ST: stent thrombosis
TLR: target lesion revascularization
