# Supplementary figures and images for: Self-reported non-adherence to P2Y12 inhibitors in patients undergoing percutaneous coronary intervention: Application of the medication non-adherence academic research consortium classification
Source: PLoS One. 2022 Feb 16;17(2):e0263180. doi: 10.1371/journal.pone.0263180 (PMC8849552; doi:10.1371/journal.pone.0263180)

**S1 Fig.** Cumulative frequency curve for non-adherence


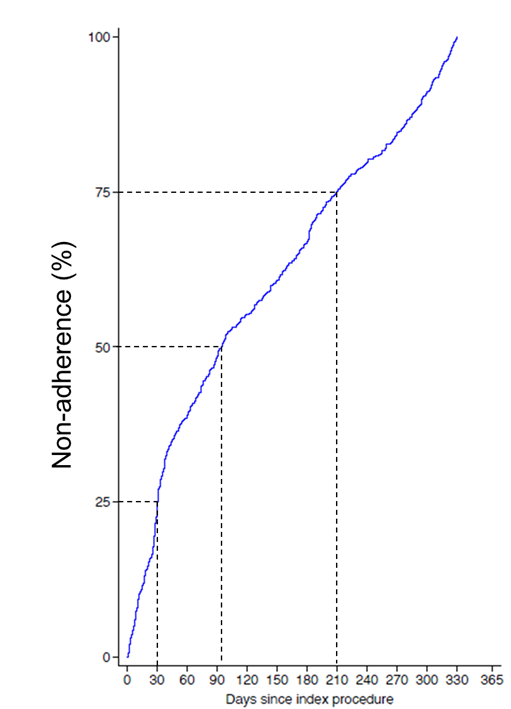

Supplement: S1 Fig — (DOCX) [file pone.0263180.s001.docx]

**S5 Fig.** Cumulative frequency curve for P2Y12 non-adherence according to PARIS category


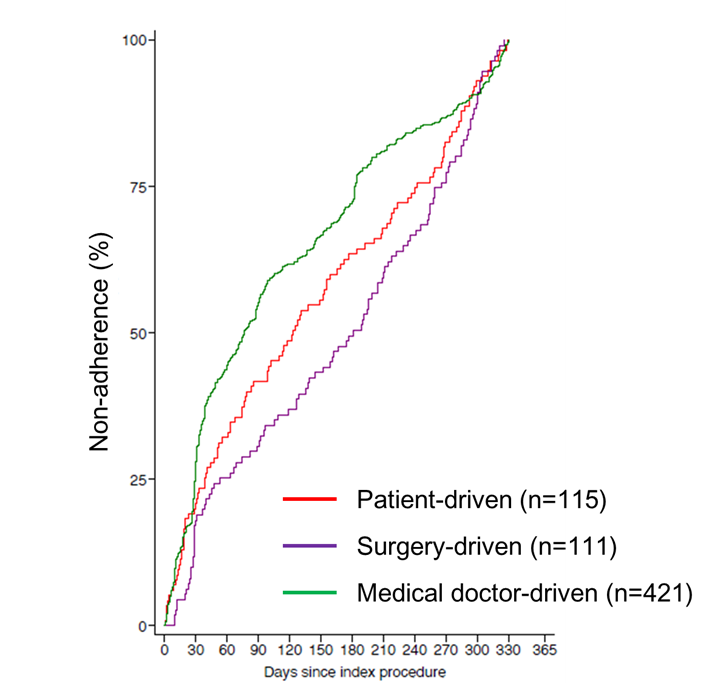

Supplement: S5 Fig — (DOCX) [file pone.0263180.s005.docx]
