# Supplementary material for: Self-reported non-adherence to P2Y12 inhibitors in patients undergoing percutaneous coronary intervention: Application of the medication non-adherence academic research consortium classification
Source: PLoS One. 2022 Feb 16;17(2):e0263180. doi: 10.1371/journal.pone.0263180 (PMC8849552; doi:10.1371/journal.pone.0263180)
Supplement: S2 Fig — (DOCX) [file pone.0263180.s002.docx]

**S2 Fig.** Multivariate Cox analysis for a composite of cardiac death, MI, and stroke according to non-adherence level


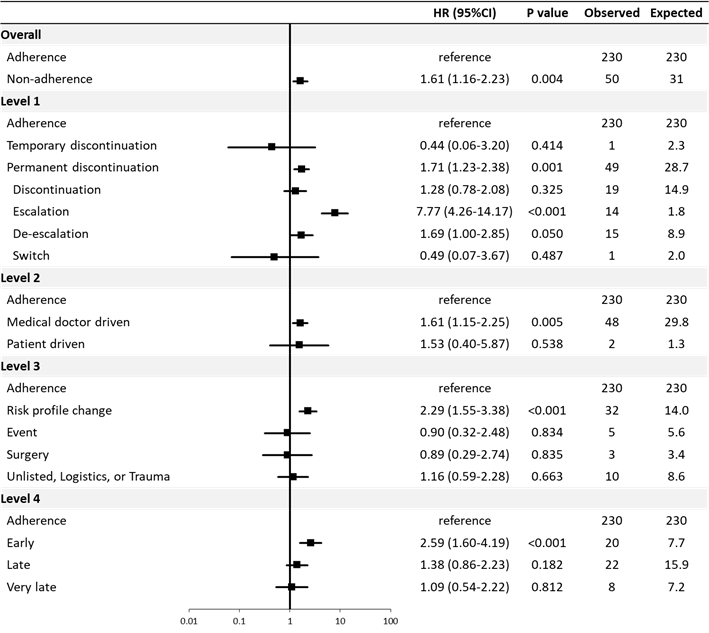


Of the study patients, 90.4% (3525 of 3896 patients) were included in the multivariable models. The following covariates were entered in the models: age, female sex, diabetes mellitus, eGFR, peripheral artery disease, myocardial infarction at presentation, cardiogenic shock, chronic obstructive lung disease, history of cancer, history of PCI, and use of new generation DES.

CI = confidence interval, DAPT = dual antiplatelet therapy, DES = drug eluting stent, eGFR = estimated glomerular filtration rate, HR = hazard ratio, PCI = percutaneous coronary intervention.
