# Supplementary material for: Self-reported non-adherence to P2Y12 inhibitors in patients undergoing percutaneous coronary intervention: Application of the medication non-adherence academic research consortium classification
Source: PLoS One. 2022 Feb 16;17(2):e0263180. doi: 10.1371/journal.pone.0263180 (PMC8849552; doi:10.1371/journal.pone.0263180)
Supplement: S3 Fig — (DOCX) [file pone.0263180.s003.docx]

**S3 Fig.** Multivariable Cox analysis for POCE at 1 year according to non-adherence levels among ACS patients


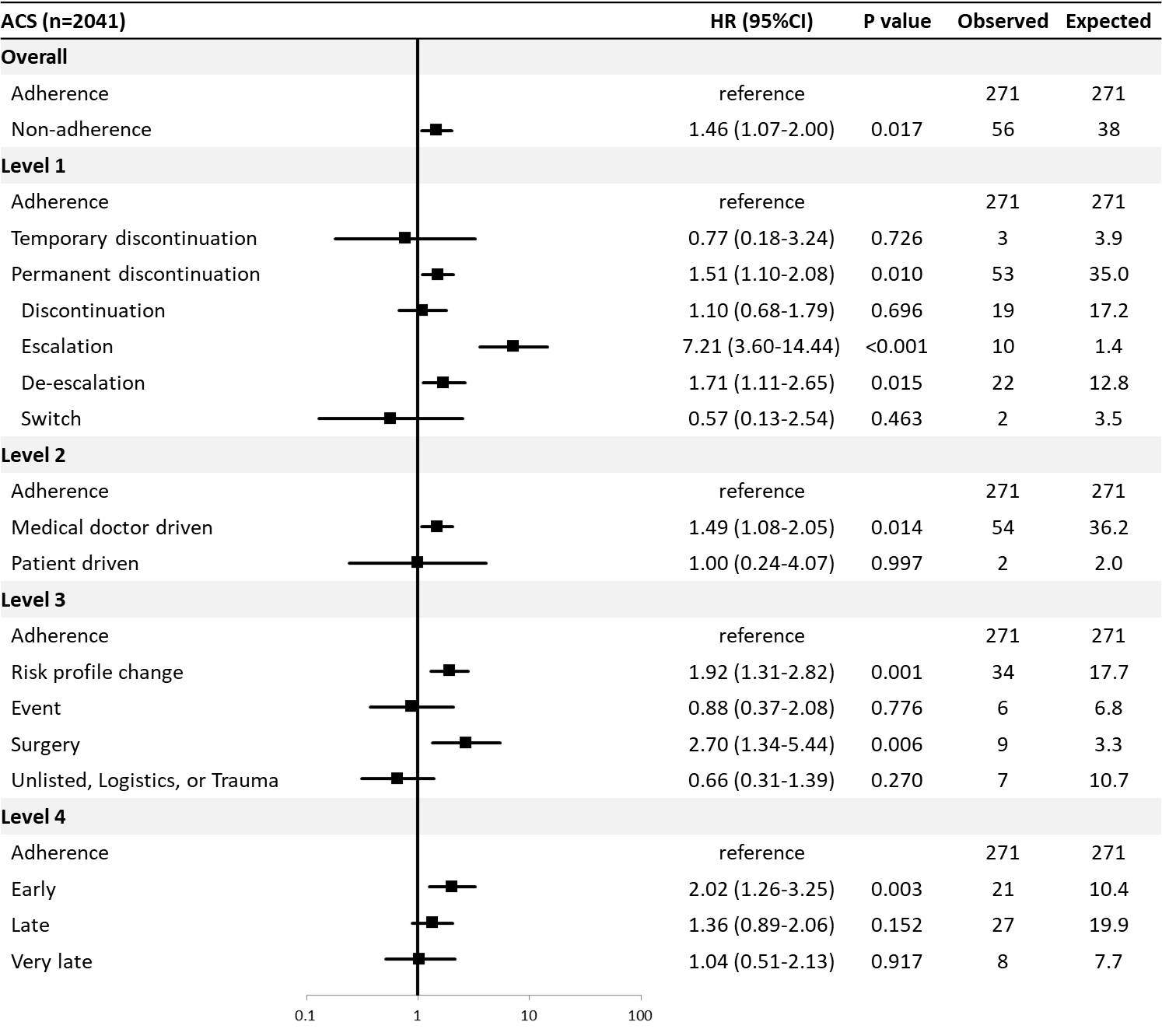


Of the study patients, 89.0% (2041 of 2293 patients) were included in the multivariable models. The following covariates were entered in the models: age, female sex, diabetes mellitus, eGFR, peripheral artery disease, myocardial infarction at presentation, cardiogenic shock, chronic obstructive lung disease, history of cancer, history of PCI, and use of new generation DES.

ACS = acute coronary syndrome, CI = confidence interval, DAPT = dual antiplatelet therapy, DES = drug eluting stent, eGFR = estimated glomerular filtration rate, HR = hazard ratio, PCI = percutaneous coronary intervention.
