# Supplementary material for: Self-reported non-adherence to P2Y12 inhibitors in patients undergoing percutaneous coronary intervention: Application of the medication non-adherence academic research consortium classification
Source: PLoS One. 2022 Feb 16;17(2):e0263180. doi: 10.1371/journal.pone.0263180 (PMC8849552; doi:10.1371/journal.pone.0263180)
Supplement: S6 Fig — (DOCX) [file pone.0263180.s006.docx]

**S6 Fig.** Kaplan-Meier cumulative event curve for POCE according to PARIS category


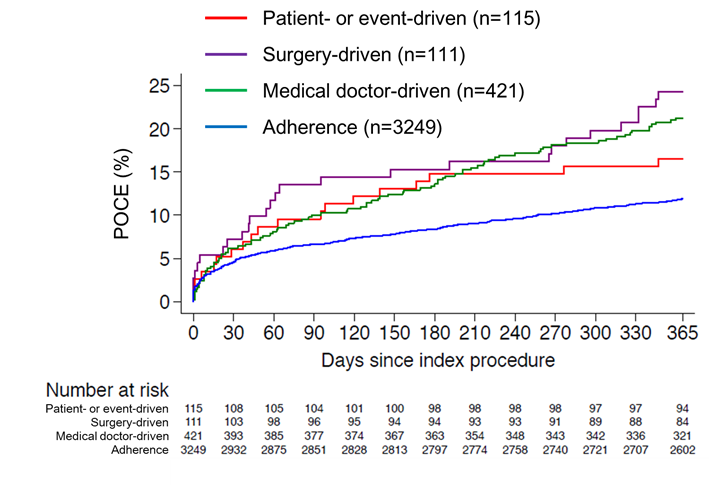


POCE = patient-oriented composite endpoint.
