# Supplementary material for: Self-reported non-adherence to P2Y12 inhibitors in patients undergoing percutaneous coronary intervention: Application of the medication non-adherence academic research consortium classification
Source: PLoS One. 2022 Feb 16;17(2):e0263180. doi: 10.1371/journal.pone.0263180 (PMC8849552; doi:10.1371/journal.pone.0263180)
Supplement: S1 Table — (DOCX) [file pone.0263180.s008.docx]

**S1 Table.** Prevalence of non-adherence according to NARC classification

| **Level 1** | **Level 1 sub** | **Level 2** | **Level 3** | **Level 4** | **Number of patients** | **%** |
| --- | --- | --- | --- | --- | --- | --- |
| Temporary | Not applicable | Medical doctor | Risk profile change | Early | 1 | 0.15 |
| Temporary | Not applicable | Medical doctor | Risk profile change | Late | 2 | 0.31 |
| Temporary | Not applicable | Medical doctor | Risk profile change | Very late | 1 | 0.15 |
| Temporary | Not applicable | Medical doctor | Events | Early | 5 | 0.77 |
| Temporary | Not applicable | Medical doctor | Events | Late | 9 | 1.39 |
| Temporary | Not applicable | Medical doctor | Events | Very late | 6 | 0.93 |
| Temporary | Not applicable | Medical doctor | Surgery | Early | 9 | 1.39 |
| Temporary | Not applicable | Medical doctor | Surgery | Late | 23 | 3.55 |
| Temporary | Not applicable | Medical doctor | Surgery | Very late | 42 | 6.49 |
| Temporary | Not applicable | Patient | Surgery | Very late | 1 | 0.15 |
| Temporary | Not applicable | Patient | Logistics | Very late | 2 | 0.31 |
| Permanent | Escalation | Medical doctor | Risk profile change | Early | 12 | 1.85 |
| Permanent | Escalation | Medical doctor | Risk profile change | Late | 16 | 2.47 |
| Permanent | Escalation | Medical doctor | Risk profile change | Very late | 7 | 1.08 |
| Permanent | De-escalation | Medical doctor | Risk profile change | Early | 29 | 4.48 |
| Permanent | De-escalation | Medical doctor | Risk profile change | Late | 53 | 8.19 |
| Permanent | De-escalation | Medical doctor | Risk profile change | Very late | 16 | 2.47 |
| Permanent | Switch | Medical doctor | Risk profile change | Early | 11 | 1.70 |
| Permanent | Switch | Medical doctor | Risk profile change | Late | 14 | 2.16 |
| Permanent | Discontinuation | Medical doctor | Risk profile change | Early | 9 | 1.39 |
| Permanent | Discontinuation | Medical doctor | Risk profile change | Late | 65 | 10.05 |
| Permanent | Discontinuation | Medical doctor | Risk profile change | Very late | 40 | 6.18 |
| Permanent | Escalation | Medical doctor | Events | Late | 1 | 0.15 |
| Permanent | De-escalation | Medical doctor | Events | Early | 4 | 0.62 |
| Permanent | De-escalation | Medical doctor | Events | Late | 4 | 0.62 |
| Permanent | De-escalation | Medical doctor | Events | Very late | 1 | 0.15 |
| Permanent | Discontinuation | Medical doctor | Events | Early | 4 | 0.62 |
| Permanent | Discontinuation | Medical doctor | Events | Late | 24 | 3.71 |
| Permanent | Discontinuation | Medical doctor | Events | Very late | 20 | 3.09 |
| Permanent | Escalation | Medical doctor | Surgery | Early | 1 | 0.15 |
| Permanent | De-escalation | Medical doctor | Surgery | Early | 4 | 0.62 |
| Permanent | De-escalation | Medical doctor | Surgery | Late | 3 | 0.46 |
| Permanent | De-escalation | Medical doctor | Surgery | Very late | 2 | 0.31 |
| Permanent | Discontinuation | Medical doctor | Surgery | Early | 3 | 0.46 |
| Permanent | Discontinuation | Medical doctor | Surgery | Late | 12 | 1.85 |
| Permanent | Discontinuation | Medical doctor | Surgery | Very late | 12 | 1.85 |
| Permanent | Escalation | Medical doctor | Unlisted | Early | 2 | 0.31 |
| Permanent | Escalation | Medical doctor | Unlisted | Late | 4 | 0.62 |
| Permanent | Escalation | Medical doctor | Unlisted | Very late | 1 | 0.15 |
| Permanent | De-escalation | Medical doctor | Unlisted | Early | 23 | 3.55 |
| Permanent | De-escalation | Medical doctor | Unlisted | Late | 23 | 3.55 |
| Permanent | De-escalation | Medical doctor | Unlisted | Very late | 4 | 0.62 |
| Permanent | Switch | Medical doctor | Unlisted | Early | 7 | 1.08 |
| Permanent | Switch | Medical doctor | Unlisted | Late | 3 | 0.46 |
| Permanent | Switch | Medical doctor | Unlisted | Very late | 2 | 0.31 |
| Permanent | Discontinuation | Medical doctor | Unlisted | Early | 3 | 0.46 |
| Permanent | Discontinuation | Medical doctor | Unlisted | Late | 24 | 3.71 |
| Permanent | Discontinuation | Medical doctor | Unlisted | Very late | 44 | 6.80 |
| Permanent | Discontinuation | Medical doctor | Unlisted | missing | 4 | 0.62 |
| Permanent | Discontinuation | Medical doctor | Trauma | Late | 1 | 0.15 |
| Permanent | De-escalation | Patient | Risk profile change | Late | 1 | 0.15 |
| Permanent | Switch | Patient | Risk profile change | Early | 1 | 0.15 |
| Permanent | Discontinuation | Patient | Events | Early | 3 | 0.46 |
| Permanent | Discontinuation | Patient | Events | Late | 8 | 1.24 |
| Permanent | Discontinuation | Patient | Events | Very late | 2 | 0.31 |
| Permanent | De-escalation | Patient | Unlisted | Early | 1 | 0.15 |
| Permanent | Discontinuation | Patient | Unlisted | Early | 4 | 0.62 |
| Permanent | Discontinuation | Patient | Unlisted | Late | 4 | 0.62 |
| Permanent | Discontinuation | Patient | Unlisted | Very late | 10 | 1.55 |

NARC = non-adherence academic research consortium.
