# Supplementary material for: Self-reported non-adherence to P2Y12 inhibitors in patients undergoing percutaneous coronary intervention: Application of the medication non-adherence academic research consortium classification
Source: PLoS One. 2022 Feb 16;17(2):e0263180. doi: 10.1371/journal.pone.0263180 (PMC8849552; doi:10.1371/journal.pone.0263180)
Supplement: S2 Table — (DOCX) [file pone.0263180.s009.docx]

**S2 Table.** Clinical outcomes occurred during adherence and non-adherence among patients with non-adherence.

|  | Non-adherence  (n=647) | | Adherence (n=3249) | P value* |
| --- | --- | --- | --- | --- |
|  | Events occurred  during adherence | Events occurred  during non-adherence |  |  |
| POCE (death, MI, revascularization, or stroke) | 54 (8.3%) | 81 (12.5%) | 367 (11.3%) | 0.437 |
| MACE (cardiac death, MI, or stroke) | 26 (4.0%) | 50 (7.7%) | 204 (6.3%) | 0.254 |
| DOCE (cardiac death, TV-MI, or TLR) | 26 (4.0%) | 43 (6.6%) | 223 (6.9%) | 0.690 |
| Death | 4 (0.6%) | 32 (4.9%) | 139 (4.3%) | 0.661 |
| Cardiac death | 3 (0.5%) | 17 (2.6%) | 88 (2.7%) | 0.739 |
| Myocardial infarction | 16 (2.5%) | 23 (3.6%) | 107 (3.3%) | 0.845 |
| Target vessel myocardial infarction | 10 (1.5%) | 14 (2.2%) | 85 (2.6%) | 0.442 |
| Periprocedural myocardial infarction | 7 (1.1%) | 0 (0%) | 47 (1.4%) |  |
| Spontaneous myocardial infarction | 9 (1.4%) | 24 (3.7%) | 61 (1.9%) | 0.007 |
| Any Revascularization | 36 (5.6%) | 45 (7.0%) | 201 (6.2%) | 0.558 |
| Target lesion revascularization | 16 (2.5%) | 26 (4.0%) | 102 (3.1%) | 0.332 |
| Stroke | 8 (1.2%) | 12 (1.9%) | 23 (0.7%) | 0.007 |
| Definite stent thrombosis | 3 (0.4%) | 5 (0.8%) | 27 (0.8%) | 0.834 |
| Any bleeding | 36 (5.6%) | 57 (8.8%) | 105 (3.2%) | <0.001 |
| BARC (3, 5) bleeding | 23 (3.6%) | 40 (6.2%) | 61 (1.9%) | <0.001 |
| BARC (2, 3, 5) bleeding | 35 (5.4%) | 54 (8.3%) | 102 (3.1%) | <0.001 |

Values are n (%).

BARC = bleeding academic research consortium, DOCE = device-oriented composite endpoints, MI = myocardial infarction, POCE = patient-oriented composite endpoints, TLR = target lesion revascularization, TV-MI = target-vessel myocardial infarction.
