# Supplementary material for: Self-reported non-adherence to P2Y12 inhibitors in patients undergoing percutaneous coronary intervention: Application of the medication non-adherence academic research consortium classification
Source: PLoS One. 2022 Feb 16;17(2):e0263180. doi: 10.1371/journal.pone.0263180 (PMC8849552; doi:10.1371/journal.pone.0263180)
Supplement: S3 Table — (DOCX) [file pone.0263180.s010.docx]

**S3 Table.** Baseline characteristics according to PARIS category

|  | Patient- or event-driven  (n=115) | Surgery-driven  (n=111) | Medical doctor-driven  (n=421) |
| --- | --- | --- | --- |
| Age (years) | 69.7 ± 12.2 | 66.7 ± 10.3 | 68.9 ± 12.1 |
| Female | 27 (23.5%) | 26 (23.4%) | 119 (28.3%) |
| Current smoker | 31 (27.2%) | 32 (29.4%) | 101 (24.4%) |
| Hypertension | 76 (66.1%) | 72 (64.9%) | 292 (69.9%) |
| Dyslipidemia | 77 (67.0%) | 71 (64.0%) | 275 (65.5%) |
| Diabetes mellitus | 20 (17.4%) | 30 (27.0%) | 95 (22.6%) |
| Insulin use | 9 (45.0%) | 12 (40.0%) | 40 (42.6%) |
| Renal failure (eGFR<60ml/min/1.73m^2^) | 32 (30.8%) | 22 (23.2%) | 123 (32.5%) |
| Anemia (men<13.0 g/dL, women<12.0 g/dL) | 33 (34.7%) | 27 (29.4%) | 98 (28.0%) |
| Chronic obstructive lung disease | 6 (5.2%) | 8 (7.2%) | 24 (5.7%) |
| History of malignancy | 11 (9.6%) | 13 (11.7%) | 56 (13.3%) |
| Peripheral arterial disease | 15 (13.0%) | 10 (9.0%) | 40 (9.5%) |
| History of Cerebrovascular Accident (Stroke/TIA) | 8 (7.0%) | 8 (7.2%) | 43 (10.2%) |
| Previous PCls | 24 (20.9%) | 23 (20.7%) | 110 (26.1%) |
| Left ventricular ejection fraction | 54.2 ± 14.2 | 54.7 ± 12.6 | 51.9 ± 14.2 |
| PRECISE-DAPT score | 20.1 ± 13.5 | 19.8 ± 10.9 | 20.6 ± 10.1 |
| Clinical indication for PCI |  |  |  |
| Chronic coronary syndrome | 36 (31.3%) | 52 (46.9%) | 165 (39.2%) |
| Unstable Angina | 7 (6.1%) | 9 (8.1%) | 12 (2.9%) |
| Non-ST elevation myocardial infarction | 40 (34.8%) | 16 (14.4%) | 134 (31.8%) |
| ST elevation myocardial infarction | 32 (27.8%) | 34 (30.6%) | 110 (26.1%) |
| Number of stents |  |  |  |
| 1 | 48 (41.7%) | 46 (41.4%) | 176 (41.8%) |
| 2 | 32 (27.8%) | 30 (27.0%) | 119 (28.3%) |
| ≥3 | 35 (30.4%) | 35 (31.5%) | 126 (29.9%) |
| Stent type |  |  |  |
| New generation drug eluting stent | 110 (95.7%) | 101 (91.0%) | 388 (92.2%) |
| 1st generation drug eluting stent | 0 (0%) | 0 (0%) | 0 (0%) |
| Bare metal stent | 4 (3.5%) | 10 (9.0%) | 31 (7.4%) |
| Total device length (mm) | 43.8 ± 28.4 | 45.7 ± 31.9 | 43.1 ± 28.5 |
| Mean stent diameter (mm) | 3.1 ± 0.5 | 3.0 ± 0.4 | 3.0 ±0.5 |
| Multivessel treatment | 32 (27.8%) | 23 (20.7%) | 117 (27.8%) |
| Bifurcation stenting | 6 (5.2%) | 8 (7.2%) | 37 (8.8%) |
| Chronic total occlusion | 5 (4.4%) | 5 (4.5%) | 19 (4.5%) |
| In-stent restenosis | 3 (2.6%) | 7 (6.3%) | 24 (5.7%) |
| Medication at discharge |  |  |  |
| Clopidogrel | 47 (40.9%) | 52 (46.9%) | 177 (42.0%) |
| Prasugrel | 20 (17.4%) | 25 (22.5%) | 73 (17.3%) |
| Ticagrelor | 48 (44.9%) | 34 (31.2%) | 171 (41.9%) |
| Statin | 106 (92.2%) | 103 (92.8%) | 375 (89.1%) |

Values are n (%) or mean±SD.

eGFR = estimated glomerular filtration rate, PCI = percutaneous coronary intervention TIA = transient ischemic attack.
