# Supplementary material for: Self-reported non-adherence to P2Y12 inhibitors in patients undergoing percutaneous coronary intervention: Application of the medication non-adherence academic research consortium classification
Source: PLoS One. 2022 Feb 16;17(2):e0263180. doi: 10.1371/journal.pone.0263180 (PMC8849552; doi:10.1371/journal.pone.0263180)
Supplement: S4 Table — (DOCX) [file pone.0263180.s011.docx]

**S4 Table.** Non-adherence according to PARIS category

|  | Patient- or event-driven  (n=115) | Surgery-driven  (n=111) | Medical doctor-driven  (n=421) |
| --- | --- | --- | --- |
| **Level 1** |  |  |  |
| Temporary discontinuation | 23 (20.0%) | 74 (66.7%) | 4 (1.0%) |
| Permanent discontinuation | 92 (80.0%) | 37 (33.3%) | 417 (99.0%) |
| Escalation | 1 (0.9%) | 1 (0.9%) | 42 (10.0%) |
| De-escalation | 11 (9.6%) | 9 (8.1%) | 148 (35.2%) |
| Switch | 1 (0.9%) | 0 (0%) | 37 (8.8%) |
| Discontinuation | 102 (88.7%) | 101 (91.0%) | 194 (46.1%) |
| **Level 2** |  |  |  |
| Medical doctor driven | 78 (67.8%) | 111 (100%) | 421 (100%) |
| Patient driven | 37 (32.2%) | 0 (0%) | 0 (0%) |
| **Level 3** |  |  |  |
| Risk profile change | 2 (1.7%) | 0 (0%) | 276 (65.6%) |
| Event | 91 (79.1%) | 0 (0%) | 0 (0%) |
| Surgery | 1 (0.9%) | 111 (100%) | 0 (0%) |
| Unlisted | 19 (16.5%) | 0 (0%) | 144 (34.2%) |
| Logistics | 2 (1.7%) | 0 (0%) | 0 (0%) |
| Trauma | 0 (0%) | 0 (0%) | 1 (0.2%) |
| Undefined | 2 (1.7%) | 0 (0%) | 276 (65.6%) |
| **Level 4** |  |  |  |
| Early | 22 (19.1%) | 17 (15.3%) | 97 (23.0%) |
| Late | 51 (44.3%) | 38 (34.2%) | 205 (48.7%) |
| Very late | 42 (36.5%) | 56 (50.5%) | 115 (27.3%) |
| Undefined | 0 (0%) | 0 (0%) | 4 (1.0%) |

Values are n (%).
