# Supplementary material for: Self-reported non-adherence to P2Y12 inhibitors in patients undergoing percutaneous coronary intervention: Application of the medication non-adherence academic research consortium classification
Source: PLoS One. 2022 Feb 16;17(2):e0263180. doi: 10.1371/journal.pone.0263180 (PMC8849552; doi:10.1371/journal.pone.0263180)
Supplement: S5 Table — (DOCX) [file pone.0263180.s012.docx]

**S5 Table.** Event rate at 1 year according to PARIS category

|  | Patient- or event-driven  (n=115) | Surgery-driven  (n=111) | Medical doctor-driven  (n=421) |
| --- | --- | --- | --- |
| POCE (death, MI, revascularization, or stroke) | 19 (16.5%) | 27 (24.3%) | 89 (21.1%) |
| DOCE (cardiac death, TV-MI, or TLR) | 10 (8.7%) | 14 (12.6%) | 45 (10.7%) |
| Death | 10 (8.7%) | 5 (4.5%) | 21 (5.0%) |
| Cardiac death | 5 (4.3%) | 3 (2.7%) | 12 (2.9%) |
| Myocardial infarction | 5 (4.3%) | 4 (3.6%) | 30 (7.1%) |
| Target vessel myocardial infarction | 3 (2.6%) | 3 (2.7%) | 18 (4.3%) |
| Periprocedural myocardial infarction | 2 (1.7%) | 2 (1.8%) | 3 (0.7%) |
| Spontaneous myocardial infarction | 4 (3.5%) | 2 (1.8%) | 27 (6.4%) |
| Any Revascularization | 5 (4.3%) | 18 (16.2%) | 58 (13.8%) |
| Target lesion revascularization | 3 (2.6%) | 11 (9.9%) | 28 (6.7%) |
| Stroke | 4 (3.5%) | 4 (3.6%) | 12 (2.9%) |
| Definite stent thrombosis | 0 (0%) | 3 (2.7%) | 5 (1.2%) |
| Any bleeding | 51 (44.3%) | 16 (14.4%) | 26 (6.2%) |
| BARC (3, 5) bleeding | 34 (29.6%) | 7 (6.3%) | 19 (4.5%) |
| BARC (2, 3, 5) bleeding | 51 (44.3%) | 12 (10.8%) | 26 (6.2%) |

Values are n (%).

BARC = bleeding academic research consortium, DOCE = device-oriented composite endpoints, MI = myocardial infarction, POCE = patient-oriented composite endpoints, TLR = target lesion revascularization, TV-MI = target-vessel myocardial infarction.
