# Supplementary material for: Self-reported non-adherence to P2Y12 inhibitors in patients undergoing percutaneous coronary intervention: Application of the medication non-adherence academic research consortium classification
Source: PLoS One. 2022 Feb 16;17(2):e0263180. doi: 10.1371/journal.pone.0263180 (PMC8849552; doi:10.1371/journal.pone.0263180)
Supplement: S1 File — (DOCX) [file pone.0263180.s013.docx]

**S1 File.** Questionnaire on the adherence to P2Y12 inhibitors

**Questionnaire on the adherence to P2Y12 inhibitors**

A prescription of «Plavix» for 12 months was given to you during the hospitalization at the Bern University Hospital at “**Date (yyyy/mm/dd)**”. It may have been replaced by “Clopidogrel-Mepha” or “Clopidogrel-Sandoz” within the time prescribed. These generics contain the same pharmaceutical substance, which has reached very good results in several large scale randomized trials. The present questionnaire’s purpose is to examine the feasibility of the drug in a “real world” setting.

Please answer the following questions even if you do not take any Plavix or Clopidogrel anymore.


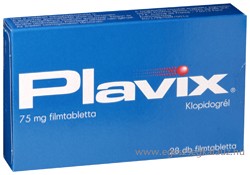

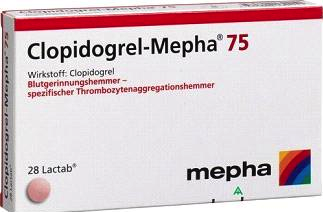


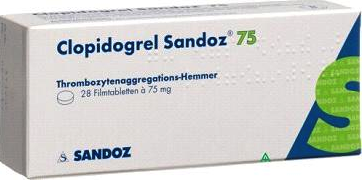


**Questionnaire regarding „Plavix“ or „Clopidogrel“**

1. Did you experience any side effects within treatment course with Plavix/Clopidogrel?

No

Yes: Dyspnea Headache Epistaxis

Nausea Rash Syncope

1. Was the treatment with Plavix/Clopidogrel paused or stopped after the treatment at the Bern University Hospital at “**Date (yyyy/mm/dd)**”

No, I have taken Plavix/Clopidogrel during the whole course of the year (Please Continue with Question 3)

Yes, Plavix/Clopidogrel was paused temporarily

Date, at which pause started: ___________________

For how many days was Plavix/Clopidogrel paused: ____________

Yes, Plavix/Clopidogrel was stopped prematurely

Stop date:__________________

If yes, who stopped and temporarily discontinued Plavix/Clopidogrel:

My doctor myself

**Please turn this page 🡪**

Please tell us the reason why Plavix/Clopidogrel was stopped prematurely or temporarily?

Because of side effects

Financial issues

I had the feeling it was unnecessary

Because of a bleeding: date______________ hospital________________

Because of an intervention: date_______ hospital__________

Other reason: __________________________________________

Was Plavix/Clopidogrel replaced by another medication?

By Efient By Brilique No new medication

1. How often did you forget to take Plavix/Clopidogrel?

Never Rarely Sometimes Often Always

1. Has it ever felt difficult to remember taking Plavix/Clopidogrel?

Never Rarely Sometimes Often Always

1. How often did you take Plavix in the foregoing month?

Always Nearly Always Most of the Time

Half of the time Less than half of the time

Thank you very much for filling out the present questionnaire and returning to us!

**
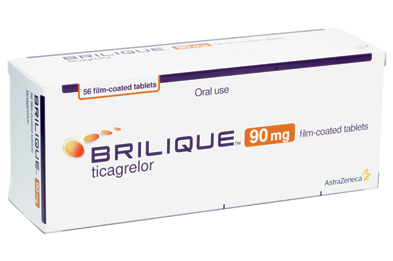
**A prescription of «Brilique» for 12 months was given to you during the hospitalization at the Bern University Hospital at “**Date (yyyy/mm/dd)**”. The present drug has reached very good results in several large scale randomized trials. The present questionnaire’s purpose is to examine the feasibility of the drug in a “real world” setting.

Please answer the following questions even if you do not take any Brilique anymore.

**Questionnaire regarding „Brilique“**

1. Did you experience any side effects within treatment course with Brilique?

No

Yes: Dyspnea Headache Epistaxis

Nausea Rash Syncope

1. Was the treatment with Brilique paused or stopped after the treatment at the Bern University Hospital at “**Date (yyyy/mm/dd)**”

No, I have taken Brilique during the whole course of the year (Please Continue with Question 3)

Yes, Brilique was paused temporarily

Date, at which pause started: ___________________

For how many days was Brilique paused: ____________

Yes, Brilique was stopped prematurely

Stop date:__________________

If yes, who stopped and temporarily discontinued Brilque:

My doctor myself

**Please turn this page 🡪**

Please tell us the reason why Brilique was stopped prematurely or temporarily?

Because of side effects

Financial issues

I had the feeling it was unnecessary

Because of a bleeding: date______________ hospital________________

Because of an intervention: date_______ hospital__________

Other reason: __________________________________________

Was Brilique replaced by another medication?

By Plavix/Clopidogrel By Efient No new medication

1. How often did you forget to take Brilique?

Never Rarely Sometimes Often Always

1. Has it ever felt difficult to remember taking Brilique?

Never Rarely Sometimes Often Always

1. How often did you take Brilique in the foregoing month?

Always Nearly Always Most of the Time Half of the time

Less than half of the time

1. How often did you forget to take Brilique in the evening?

Never Rarely Sometimes Often Always

1. Has it ever felt difficult to remember taking Brilique in the evening?

Never Rarely Sometimes Often Always

1. How often did you take Brilique in the foregoing month in the evening?

Always Nearly Always Most of the Time Half of the time

Less than half of the time

Thank you very much for filling out the present questionnaire and returning to us!

**
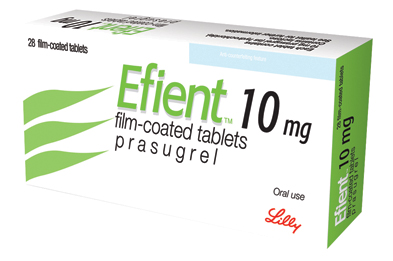
**A prescription of «Plavix» for 12 months was given to you during the hospitalization at the Bern University Hospital at “**Date (yyyy/mm/dd)**”. The present drug has reached very good results in several large scale randomized trials. The present questionnaire’s purpose is to examine the feasibility of the drug in a “real world” setting.

Please answer the following questions even if you do not take any Efient anymore.

**Questionnaire regarding „Efient“**

1. Did you experience any side effects within treatment course with Efient?

No

Yes: Dyspnea Headache Epistaxis

Nausea Rash Syncope

1. Was the treatment with Efient paused or stopped after the treatment at the Bern University Hospital at “**Date (yyyy/mm/dd)**”

No, I have taken Efient during the whole course of the year (Please Continue with Question 3)

Yes, Efient was paused temporarily

Date, at which pause started: ___________________

For how many days was Efient paused: ____________

Yes, Efient was stopped prematurely

Stop date:__________________

If yes, who stopped and temporarily discontinued Efient:

My doctor myself

**Please turn this page 🡪**

Please tell us the reason why Efient was stopped prematurely or temporarily?

Because of side effects

Financial issues

I had the feeling it was unnecessary

Because of a bleeding: date______________ hospital________________

Because of an intervention: date_______ hospital__________

Other reason: __________________________________________

Was Efient replaced by another medication?

By Plavix/Clopidogrel By Brilique No new medication

1. How often did you forget to take Efient?

Never Rarely Sometimes Often Always

1. Has it ever felt difficult to remember taking Efient?

Never Rarely Sometimes Often Always

1. How often did you take Efient in the foregoing month?

Always Nearly Always Most of the Time

Half of the time Less than half of the time

Thank you very much for filling out the present questionnaire and returning to us!
